# Succinimide Derivatives as Antioxidant Anticholinesterases, Anti-*α*-Amylase, and Anti-*α*-Glucosidase: In Vitro and In Silico Approaches

**DOI:** 10.1155/2022/6726438

**Published:** 2022-07-30

**Authors:** Osama M. Alshehri, Mater H. Mahnashi, Abdul Sadiq, Rehman Zafar, Muhammad Saeed Jan, Farhat Ullah, Mohammed Ali Alshehri, Saleh Alshamrani, Elhashimi E. Hassan

**Affiliations:** ^1^Department of Clinical Laboratory Sciences, College of Applied Medical Sciences, Najran University, Najran, Saudi Arabia; ^2^Department of Pharmaceutical Chemistry, College of Pharmacy, Najran University, Najran, Saudi Arabia; ^3^Department of Pharmacy, Faculty of Biological Sciences, University of Malakand, Chakdara, KP 18000 Dir (L), Pakistan; ^4^Riphah Institute of Pharmaceutical Sciences, Riphah International University, Islamabad 44000, Pakistan; ^5^Department of Pharmacy, University of Swabi, Swabi, KP, Pakistan; ^6^Department of Medical Genetics, Collage of Applied Medical Sciences, Najran University, Najran, Saudi Arabia

## Abstract

Based on the diverse pharmacological potency and the structural features of succinimide, this research considered to synthesize succinimide derivatives. Moreover, these compounds were estimated for their biological potential in terms of anti-diabetic, anti-cholinesterase, and anti-oxidant capacities. The compounds were synthesized through Michael addition of various ketones to *N-*aryl maleimides. Similarly, the MOE software was used for the molecular docking study to explore the binding mode of the potent compounds against different enzymes. In the anti-cholinesterase activity, the compounds **MSJ2** and **MSJ10** exhibited outstanding activity against acetylcholinesterase (AChE), i.e., 91.90, 93.20%, and against butyrylcholinesterase (BChE), i.e., 97.30, 91.36% inhibitory potentials, respectively. The compounds **MSJ9** and **MSJ10** exhibited prominent *α*-glucosidase inhibitory potentials, i.e., 87.63 and 89.37 with IC_50_ value of 32 and 28.04 *μ*M, respectively. Moreover, the compounds **MSJ2** and **MSJ10** revealed significant scavenging activity against DPPH free radicals with IC_50_ values of 2.59 and 2.52, while against ABTS displayed excellent scavenging potential with IC_50_ values 7.32 and 3.29 *μ*M, respectively. The tentative results are added with molecular docking studies in the active sites of enzymes to predict the theoretical protein-ligand binding modes. Further detailed mechanism-based studies in animal models are essential for the in vivo evaluation of the potent compound.

## 1. Introduction

Diseases adversely affect the health and well-being of human population [1]. In the 21^st^ century, non-communicable diseases have become the main public concern [2]. Nature gave unexhaustive bounties to explore, experiment, analyze, and utilize to cure and treat human and animal ailments successfully such as hepatitis, diabetes, malaria, jaundice, inflammation, skin disorders, and depression [3, 4]. Recently, extensive research is still ongoing to find remedies to many concerning pathological conditions including diabetes, hepatitis, and cancer, which are not only effective in complete amelioration of the diseased condition but are also safe and economical [5, 6]. Day by day, there is an increasing demand for medicine and treatment of many diseases [7]. Continual use of some drugs may lead to side effects or drug interactions when used to treat such ailments [8]. There is a need to look for new purpose-based research work, using their therapeutic values because various drugs cause various side effects [9], e.g., sulfonamides inhibit the metabolism or excretion of anti-diabetic drug sulfonylureas, resulting in hypoglycemia, while rifampicin increases their metabolism to reduce their hypoglycemic effect [10].

Diabetes mellitus (DM) can cause illness for prolonged time due to which the premature mortality ratio is high as compared to HIV-AIDS with approximately one death in every ten seconds across the globe [11]. This particular metabolic disease is characterized by defects in insulin secretion with subsequent effects displayed in the spectrum of hyperglycemia [12]. Diabetes mellitus can worsen in a few years due to which it is a main threat for human health worldwide [13]. In developed countries, diabetes is the fourth to fifth major cause of mortality, and signifies one of the most common non-communicable diseases worldwide [14, 15]. The current estimate revealed that among 150 to 220 million people were affected in 2010, with a forecast of 300 million individuals in 2025 worldwide [16]. Developing countries had maximum increases in the last few years. It has been estimated that there will be the largest number of diabetic subjects in the world by 2025 [17, 18]. DM is one of the major killers in Asian and Western Pacific peoples as described by the World Health Organization [19].

Free radicals have been involved in several diseases such as in cancer, liver cirrhosis, diabetes mellitus, and atherosclerosis [20], and those compounds which can easily remove these free radicals have great potential in amending the subject disease progression [21]. In diabetes mellitus, free radicals production like ROS (reactive oxygen species) is increased in all tissues from protein glycosylation and glucose auto-oxidation due to persistence hyperglycemia [22]. Different studies have shown that DM is mostly associated with oxidative stress, as a result of high production of ROS [23], superoxide radicals (O_2_), hydroxyl radicals (OH), hydrogen peroxide (H_2_O_2_), and/or decrease in the anti-oxidant defense system. In the pathogenesis of DM (diabetes mellitus), the effect of oxidative stress is not only due to the presence of free radicals but also due to decreased anti-oxidant enzyme, glucose auto-oxidation, non-enzymatic protein glycosylation, and formation of peroxides [24, 25].

In the progression of Alzheimer's disease, oxidative stress is one of the initial events before the formation of AD plaques; to counteract this effect with interrelated factors is to raise the conation of ACH through the inhibition of AChE (acetylcholinesterase) and BChE (butyrylcholinesterase), which are responsible for the breakdown of acetylcholine in the synaptic clefts [26]. On the other hand, cholinesterase inhibitors have proven to be inadequate to stop or slow down the neurodegenerative process but partially compensate the lost cognitive functions [26]. It is proposed that the treatment of Alzheimer's would benefit with the use of multipotent drugs with cholinesterase (AChE or BChE) and *β*-secretase activities as well oxidative stress-reducing capability [27].

Succinimides have been observed to play a significant role in therapeutic strategies [28]. However, the use of succinimide derivatives is supposed to be a virtuous way to improve metabolic stability and pharmacokinetic properties. Various nitrogen-containing derivatives use succinimide derivatives as the building blocks. Reagents are required for the irregular addition [14, 29]. Succinimides are the well-known class of compounds possessing anti-Alzheimer potential through dual inhibitory pathways. They follow the cholinesterase inhibition at one side and behave as antioxidants on the other [30]. These classes of compounds also have been reported for other pharmacological activities. Succinimide has the basic nuclei of pyrrolidine-2,5-dione which is a five-member heterocyclic ring having nitrogen as the heteroatom and two carbonyl groups attached as functional groups. This basic skeleton can be altered to form carbon- or nitrogen-substituted derivatives with various aryl or alkyl groups that can formulate potential drug molecules [30]. The various synthetic and natural drugs commonly employed in neurological diseases like Alzheimer's possess an aromatic ring, a nitrogen atom, or a carbonyl group in their structure. Likewise, compounds used as free radical scavengers should have electron-rich moieties like hydroxyl groups in conjugation with their structures. This is the reason why these synthesized succinimide derivatives have been tested for enzyme inhibitory potential against cholinesterase enzymes including acetylcholinesterase and butyrylcholinesterase, *α*-amylase, *α*-glucosidase, and anti-oxidant activities. The as acquired derivatives were then *in vitro* screened in enzymes assays for AChE, BChE, ABTS, DPPH, alpha glucosidase, and alpha amylase inhibition. The enzyme interactions were then further validated using molecular docking studies.

## 2. Material and Methods

All of the chemicals, solvents, and drugs used in the analyses were obtained from Sigma Aldrich's local seller. Tween 80 (CAS no; 9005-65-6), Alloxan (CAS no: 50-71-5), E.A (CAS no; 141-78-6), maleimides (CAS no; 541-59-3), chloroform (CAS no; 67-66-3), silica gel (CAS no; 7631-86-9), n-hexane (CAS no; 110-54-3), *α*-glucosidase (CAS no; 9001-42-7), KOH (CAS no; 1310-58-3), *α*-amylase (CAS no; 9000-90-2), creatinine (CAS no; 67-7-5), phosphate buffer, glibenclamide, and other important chemicals were purchased from the standard quality supplier.

### 2.1. Compounds' Synthesis

The synthesis of the compounds (**MSJ 1—10**) was carried out by organocatalytic Michael addition. Various cyclic and acyclic ketones were added to N-substituted aryl-maleimides. Initially, 2.0 equivalent of ketone was added to chloroform (1 M) in the presence of catalytic amounts of L-isoleucine and KOH (each 0.2 equivalent) in a small reaction vial. This was stirred for a short time to produce the nucleophilic enamine species of the respective ketones. Afterwards, the maleimides were added to the respective reaction as the limiting reagent (1 equivalent) to precede the reaction. The reaction was routinely checked by TLC analysis. The reaction was quenched with distilled water when the limiting reagent disappeared on the TLC analysis. The upper layer (organic) was separated 3 times from the water layer. The materials were dried and subjected to flash chromatography for the isolation of pure compounds [31].

### 2.2. Anti-Oxidant Activity

#### 2.2.1. DPPH Radical Scavenging Assay

A previously reported method for DPPH radical scavenging activity was used to determine the anti-oxidant activity of the synthetic compounds with insignificant modification [12]. A solution of DPPH, e.g., (50 *µ*L of 1.0 × 10^−3^ M), was prepared freshly and then added to CH_3_OH (methyl alcohol). A solvent like methanol was used as a control group. The mixture was then incubated for 30 minutes at 25°C. A spectrophotometer is used to calculate the DPPH free radicals at specific wavelength like 517 nm. After the incubation period, Trolox (drug) was used as the control group (positive). The anti-oxidant activity was determined using the following formula:

(1) $$\begin{array}{c} \text{Percent Inhibition}=\left[\frac{\text{Absorbance}\left(\text{control}\right)-\text{Absorbance}\left(\text{test sample}\right)}{\text{Absorbance}\left(\text{control}\right)}\right]\times 100. \end{array}$$

#### 2.2.2. ABTS Radical Scavenging Assay

A previously reported method for ABTS radical scavenging assay was used to determine the anti-oxidant activity of the synthetic compounds with negligible modification [32]. The stock solution was prepared, e.g., 7 mM ABTS solution and 2.4 mM of potassium persulfate solution, and then mixed in equal amounts for about 16 hours. The stock solution was then diluted with methyl alcohol. Using a spectrophotometer, the absorption (0.7 ± 0.02) units at 734 nm wavelength were identified. In this assay, the solution was prepared freshly. The ABTS solution (150 *µ*l) was mixed with the synthesized compounds (50 *µ*l) and then reserved in darkness for about 10 minutes. The same procedure was repeated 3 times and then the results were verified using mean ± standard deviation. A solvent, e.g., methanol, was used as blank and the standard (butyl hydroxytoluen in methanol) were run at the same time. The absorbance was checked at a wavelength of 734 nm by utilizing a microplate reader.

#### 2.2.3. Anti-Cholinesterase Assay

In Ellman's test, enzymes like (BChE) butyrylcholinesterase from equine serum and (AChE) acetylcholinesterase from electric eel were designated for studying the enzyme inhibitory capacity of the new synthesized compounds [33, 34]. This test is performed on hydrolyzed butyrylthiocholine or acetylthiocholine iodide through equivalent enzymes; as a result, 5-thio-2-nitrobenzoate anion was formed with DTNB and gives yellowish color compounds that were observed by a spectrometer alongside reaction time.

### 2.3. Preparation of Solutions

Synthetic compounds in concentration ranging from 31.25 to 500 *µ*g/ml, KH_2_PO_4_ (13.6 g/L) and K_2_HPO_4_ (17.4 g/L), were dissolved in 0.1 M phosphate buffer solution. After mixing, they were combined in 6 and 94% ratio, to make 0.1 M and 8.0 phosphate buffer solutions having 0.1 pH. Potassium hydroxide was used to change the pH. The freshly prepared buffer pH 0.8, AChE (518 U/mg solid), and BChE (7–16 U/mg) were diluted to achieve final absolute concentrations of 0.01 U/ml and 0.03 U/ml, respectively. In distilled water, BChI (0.0005 M), DTNB iedithio bis-nitrobenzoic acid solution (0.0002273 M), and AChI were prepared and then kept in the refrigerator [35].

### 2.4. Spectroscopic Analysis

In this assay, introduce 5 *μ*l of the enzyme solution into a cuvette which has 205 *μ*l of the synthesized compound solution; then, add 5 *μ*l of the DTNB iedithio bis-nitrobenzoic acid reagent. After that, the substrate solution (5 *μ*l) was added into the solution present in mixture form and was held in a water bath for 15 minutes at 30°C. Absorbance was measured at 412 nm using a double beam spectrophotometer [24]. In this assay, galanthamine (cholinesterase inhibitor) was used as a positive control drug while all components except the compound have been used as the negative control group. At 30°C, the absorbance and the time of the reaction were measured at 30°C for four minutes and repeated three times. Finally, the activity of the control groups, enzymes, and test sample were assessed using the following formula (*V* = Abs/*t*)

(2) $$\begin{array}{c} \%\text{of enzyme inhibition}=100-\%\text{activity of enzyme,}\\ \%\text{of enzyme activity}=100\times \frac{V}{V_{\max}},\\ V_{\max}=\text{enzyme activity in the absence of potent inhibitory drug.} \end{array}$$

### 2.5. Anti-Diabetic Assay

#### 2.5.1. *α*-Glucosidase Activity

In this assay, glucopyranoside is added to the solvent (phosphate buffer). Various concentrations of the synthetized compounds like 500, 250, 125, 62.5, and 31.25 *µ*g/ml were used for preparing the sample solutions. Glucosidase in distilled water (0.5 *µ*g/ml) was added to the above mixture. This mixture was then incubated for 20 minutes at 37°C [36]. After the incubation period, the reaction mixture was ceased by the addition of hydrochloric acid. The color intensity was determined at 540 nm wavelength using a spectrophotometer. The formula which was used to determine percentage inhibition is:

(3) $$\begin{array}{c} \text{Percent Inhibition}=\frac{\text{Absorbance}\left(\text{Control}\right)-\text{Absorbance}\left(\text{Sample}\right)}{\text{Absorbance }\left(\text{Control}\right)}\times 100. \end{array}$$

#### 2.5.2. *In Vitro* Assay of *α*-Amylase Activity

In this assay, previously documented protocols were used [37]. For preparing the sample solution, alpha amylase was mixed with the solvent (phosphate buffer) and also various concentrations of the synthesized compounds (500 *µ*g/ml, 250 *µ*g/ml, 125 *µ*g/ml, 62.5 *µ*g/ml, and 31.25 *µ*g/ml) were mixed in this solution. Starch solution was then mixed with the above solution and then incubated for about 20 minutes at 37°C. After incubation, the reactant mixture was then kept in a water bath for some time at 100°C. Color intensity was measured using a microplate reader at 656 nm. To determine percentage inhibition, the following formula was used:

(4) $$\begin{array}{c} \text{Percent Inhibition}=\frac{\text{Absorbance}\left(\text{Control}\right)-\text{Absorbance}\left(\text{Sample}\right)}{\text{Absorbance }\left(\text{Control}\right)}\times 100. \end{array}$$

#### 2.5.3. *In Silico* Docking Studies

Docking studies of the synthesized compounds were carried out to analyze the stabilizing interactions of all synthesized compounds **MSJ (1-10)** inside the protein pockets of selected targeted macromolecules. Docking studies were performed through Autodock Vina 1.1.2 interlinked with PyRx Software that has excellent authenticity to perform. The Pdb IDs of all targeted protein moieties (AChE-1EVE, BChE-4BDS, *α*-amylase-5U3A, and *α*-glucosidase-5NN3) were downloaded from an online data bank server https://www.rcsb.org/pdb and saved in the Pdb format. LGA (Lamarckian genetic algorithm) and the empirical energy-free function were employed for docking scoring through PyRx. The three-dimensional structures of all the synthesized compounds were generated through the latest version 20.0.0.41 of PerkinElmer Chem Draw professional software. Files were saved in the mol. Format. Furthermore, these structures were converted to the Pdb format through BioVia Discovery Studio Visualizer 20.0 after the addition of polar hydrogen. Meanwhile, the Pdb IDs of all targeted proteins were purified by removing the co-crystallized ligands and adding polar hydrogen, in addition to energy minimization. Docking runs were generated by adjusting the grid box with dimensions (Å) X: 52.21, Y: 51.98, Z: 48.12. Out of top-10 postures generated, the one having a greater negative binding energy was selected for each of the synthesized compounds and analyzed to understand the nature of the binding interactions.

## 3. Results

### 3.1. Chemistry of the Compounds

**Figure 1:**
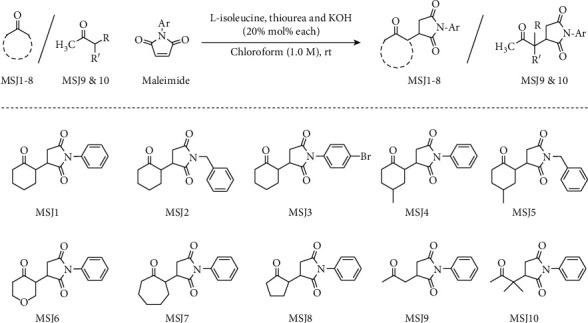
Structures of the synthesized compounds (**MSJ 1—10**).

Ten different ketone derivatives of succinimides (**MSJ 1—10**) have been synthesized and evaluated in this study as shown in Figure 1. The compounds **MSJ1** and **MSJ2** have been synthesized with cyclohexanone additions to N-phenyl and N-benzylmaleimides with isolated yields of 90 and 69%, respectively. **MSJ1** was a white solid while **MSJ2** was half white. The compounds **MSJ4** and **MSJ5** are the extended structures/modifications of **MSJ1** and **2** where a 4-methylcyclohexanone has been used in the synthesis of both. The isolated yields for MSJ4 and 5 were 79 and 63%, respectively. MSJ4 was a white solid while MSJ5 was yellowish in color. The compound **MSJ3** is synthesized by reacting cyclohexanone with N-(4-bromo)phenylmaleimide with 75% isolated yield and with yellowish color. The compound **MSJ6** has been synthesized with a derivative of cyclohexanone having a heteroatom (oxygen) at the para position. The compound was half white in color with 73% isolated yield. Similarly, in structures **MSJ7** and **8**, the ketone ring is increased and decreased by a methylene unit, respectively. The isolated yields of compounds **MSJ7** (white) and **8** (yellowish) were 78 and 74%, respectively. The last two compounds (**MSJ9** and **MSJ10**) have been synthesized with acyclic ketones, i.e., acetone (for **MSJ9**) and 3-methyl-2-butanone (for **MSJ10**). Both of these compounds were yellowish in color with isolated yields of 76 (**MSJ9**) and 70% (**MSJ10**). The 1H and 13C NMR spectra are provided in the supplementary material, Figures S1–S10.

### 3.2. Results of the Anti-Oxidant Assays

In the DPPH scavenging assay, the succinimide derivatives **MSJ (1–10)** were tested at different concentrations of 31.25, 62.5, 125, 250, and 500 *μ*mol/mL, respectively. In this assay, the most potent compound was **MSJ10** which caused a percent radical scavenging of 93.03 ± 0.48, 90.90 ± 0.48, 85.79 ± 0.63, 79.67 ± 0.61, and 75.69 ± 0.77 (IC_50_ 2.52 *µ*M), the second highest activity was displayed by **MSJ2** causing 86.47 ± 0.70, 84.47 ± 0.46, 81.50 ± 0.61, 78.23 ± 0.44, and 73.45 ± 0.65 (IC_50_ 2.59 *µ*M), respectively. All the other compounds like **MSJ1**, **MSJ3**, **MSJ4**, **MSJ5**, **MSJ6**, **MSJ7**, **MSJ8,** and **MSJ9** also exhibited significant results causing IC_50_ 34.57 *µ*M, 33.47 *µ*M, 31.96 *µ*M, 39.01 *µ*M, 44.86 *µ*M, 31.44 *µ*M, 29.23 *µ*M, and 53.02 *µ*M, respectively. The standard drug, ascorbic acid, displayed 98.65 ± 1.32, 93.56 ± 0.45, 91.52 ± 0.66, 88.22 ± 1.28, and 86.42 ± 0.43 at various concentrations with IC_50_ 6.25 *µ*M as shown in Table 1. The percent inhibition values at concentrations ranging from the 500 to 31.25 *μ*mol/ml of the various synthesized compounds are exhibited in Figure S11.

In comparison to ABTS, the compounds exhibited significant DPPH free radical scavenging activity. Using this assay, all the 10 compounds were tested at concentrations (31.25–500 *µ*mo/mL). In the DPPH anti-radical assay, again, the compounds **MSJ10** and **MSJ2** had the highest activity with percent inhibitions of 93.03 ± 0.48, 90.90 ± 0.48, 85.79 ± 0.63, 79.67 ± 0.61, and 75.69 ± 0.77 (IC_50_ 3.29 *µ*M), and 90.09 ± 0.32, 88.67 ± 1.20, 83.40 ± 0.25, 78.58 ± 1.12, and 74.65 ± 1.34 (IC_50_ 7.32 *µ*M) correspondingly (Table 1). Ascorbic acid (positive control) inhibition was (IC_50_ 4.66 *µ*M). All the other compounds also displayed good to moderate activity (Figure S12).

### 3.3. Results of the Anti-Cholinesterase Assay

The results of the anti-cholinesterase assay of the tested compounds as well as the positive control galantamine IC_50_ values are summarized in Table 2. The compound **MSJ10** exhibited outstanding anti-cholinesterase potential against both AChE and BChE. The percent anti-AChE and anti-BChE potentials displayed by the tested compound were very comparable 93.20 ± 0.10, 90.09 ± 0.32, 88.67 ± 1.20, 83.40 ± 0.25, 78.58 ± 1.12 and 91.36 ± 0.39, 87.15 ± 1.07, 83.00 ± 0.44, 78.26 ± 0.43, 73.89 ± 0.49 to that of the galantamine which is used as the standard drug. The IC_50_ value of the test compound against AChE and BChE was deliberated to be 4.97 and 10.72 *μ*M, while the positive control displayed IC_50_ values of 0.762 *μ*g/mL against AChE and 6.31 *μ*M against BChE, respectively. This shows the effectiveness of the synthesized succinimide derivatives against Alzheimer's disease. All the other compounds also exhibited well to moderate activity against both AChE and BChE. The anti-AChE scavenging potential of the tested compounds were in an ascending order of MSJ10 > MSJ2 > MSJ7 > MSJ1 > MSJ8 > MSJ4 > MSJ5 > MSJ6 > MSJ3 > MSJ9, respectively (Figures S13 and S14).

### 3.4. Results of the Anti-Diabetic Assay

#### 3.4.1. *α*-Glucosidase Inhibitory Assay

As *α*-glucosidase is an enzyme responsible for diabetes, it has been used for the assessment of the anti-diabetic potential of the subject compounds. Analysis of the tested samples against *α*-glucosidase revealed that the highest scavenging effect is shown by compound **MSJ10**, which shows 89.37 ± 0.54, 84.44 ± 0.50, 77.51 ± 0.72, 72.28 ± 0.61, and 67.46 ± 0.62 activity at concentrations of 500, 250, 125, 62.5, and 31.25 *µ*mol/mL, respectively (Figure S15) with the IC_50_ value 28.04 *µ*M. The standard drug acarbose exhibited 94.40 ± 0.03, 85.03 ± 2.16, 80.90 ± 1.11, 76.44 ± 0.28, and 71.22 ± 0.47% inhibition at 500-31.25 *µ*mol/mL, respectively, with 9.76 *µ*M IC_50_ value. The lowest *α*-glucosidase scavenging activity was recorded for **MSJ6** possessing the IC_50_ value 155.59 *µ*M. The second highest activity was displayed by **MSJ9** with the IC_50_ value 32 *µ*M as shown in Table 3.

#### 3.4.2. *α*-Amylase Inhibition Assay

In the *α*-amylase inhibition assay, the highest activity was shown by **MSJ10**. It displayed 86.91 ± 1.30, 81.26 ± 1.27, 76.00 ± 0.30, 71.54 ± 0.50, and 68.76 ± 0.58 percent inhibitions at concentrations from 500 to 31.25 *µ*mol/mL with the IC_50_ of 16.62 *μ*M. The compound **MSJ9** showed the second highest activity, resulting in 94.40 ± 0.03, 85.03 ± 2.16, 80.90 ± 1.11, 76.44 ± 0.28, and 71.22 ± 0.47 with an IC_50_ of 27.24 *μ*M. Acarbose as a standard drug showed an activity of 91.90 ± 0.96, 87.08 ± 0.47, 82.40 ± 0.20, 77.61 ± 0.43, and 75.45 ± 0.90 percent inhibition (Figure S16) with an IC_50_ value of 3.86 *μ*M against *α*-amylase (Table 3). All the other tested compounds in this assay displayed good to moderate *α*-amylase inhibition potential.

### 3.5. Results of In Silico Molecular Docking

Docking studies acquired a convinced position in scrutinizing and calculating the right way of ligand binding into active site (Ref). They provide an accurate approach of identification of ligand behavior inside the binding pockets of macromolecules (Ref). All the synthesized compounds **MSJ1** to **MSJ10** were docked with targeted proteins: acetylcholinesterase, butyrylcholinesterase, *α*-amylase, and *α*-glucosidase whose active sites were identified through co-crystallized ligands. The resultant binding energies of best postures are mentioned in Table 4.

**Figure 2:**
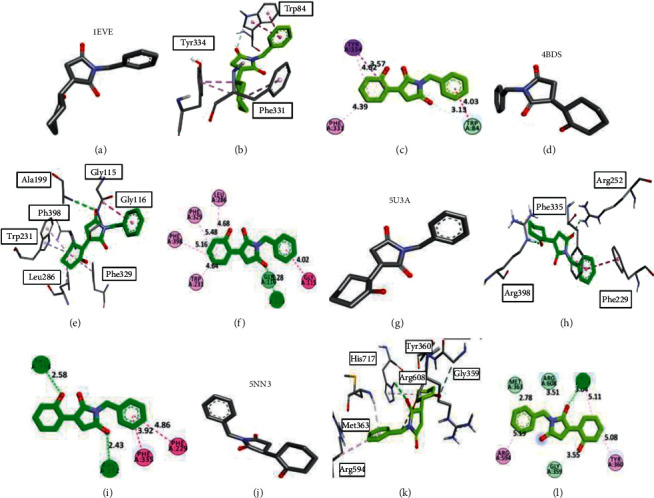
Docking 3-dimensional and 2-dimensional poses of ligand **MSJ2** inside the binding pockets of the targeted proteins. (a–c) indicate the best binding postures with acetylcholinesterase, (d–f) with butyrylcholinesterase, (g–i) with *α*-amylase, and (j–l) with *α*-glucosidase.

All of the synthesized compounds performed satisfactorily with the targeted macromolecules, but the results were excellent in the case of **MSJ2** and **MSJ10,** showing increased binding affinities with the protein moieties. These results were validated through comparison with co-crystallized ligands of each targeted macromolecule. The results were compared in terms of RMSD values, binding posture of ligands, and interactions like conventional hydrogen bonds, pi-pi bonds, pi-sigma interaction, carbon-hydrogen bonds, and others appearing. Regarding the binding behavior of **MSJ2** with acetylcholinesterase, it gave −8.7 Kcal/mol binding energy in its best pose, while in the case of other targets, it gave −8.9 Kcal/mol, −8.2 Kcal/mol, and −8.5 Kcal/mol when interacting with butyryl cholinesterase, *α*-amylase, and *α*-glucosidase, respectively, a satisfactory comparison with in vitro results. All the visual parameters have been described in Figure 2.

When interacting with acetylcholinesterase, the cyclohexanone side chain established strong pi-sigma bond with TYR A: 334, while the aromatic ring observed a carbon-hydrogen bond with TRP A: 84. This interaction becomes more prominent in the case of butyrylcholinesterase, in which the cyclohexanone formed four alkyl and pi-alkyl linkages with TRP A: 231, LEU A: 286, PHE A: 329, and PHE A: 398. Amide pi-stacked was observed with GLY A: 115. One strong conventional hydrogen bond with ALA A: 199 further stabilized the linkages. The other prominent amino acid residues at the active site that were involved in the interaction in the case of *α*-amylase and *α*-glucosidase were PHE A: 229, PHE A: 252, PHE A: 335, ARG A: 398, TYR A: 360, ARG A: 594, and HIS A: 717.

**Figure 3:**
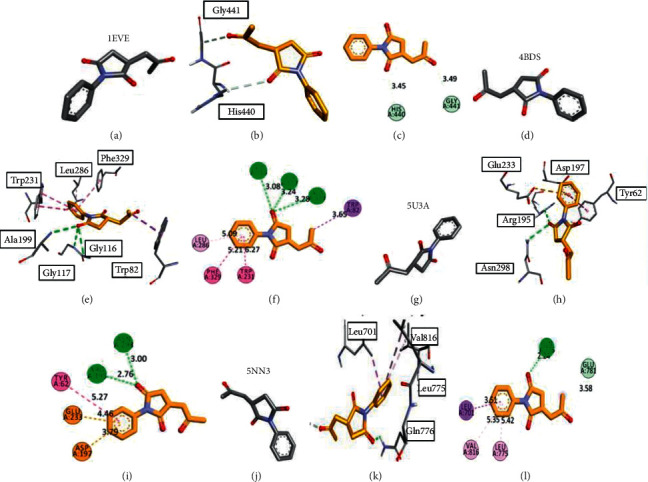
Docking 3-dimensional and 2-dimensional poses of ligand **MSJ9** inside the binding pockets of the targeted proteins. (a–c) indicate the best binding postures with acetylcholinesterase, (d–f) with butyrylcholinesterase, (g–i) with *α*-amylase, and (j–l) with *α*-glucosidase.

Figure 3 represents the behavior of MSJ9 with all targeted proteins. This compound was stabilized inside the binding pocket through conventional hydrogen bonds. It formed carbon-hydrogen bonds with HIS A: 440 and GLY A: 441 in the case of acetylcholinesterase, with the best pose giving a binding affinity of −6.6 Kcal/mol. This ligand gave more interesting linkages with GLY A: 116, Gly A: 117, and ALA A: 199 in butyrylcholinesterase through three conventional hydrogen bonds. The prominent linkage was butan-2-one side chain with TRP A: 82 that stabilized this linkage further. Moreover, this ligand formed conventional hydrogen bonds with GLN A: 776 in *α*-amylase PDB ID: 5U3A with a bond length of 2.24 Å. The aromatic ring in this ligand formed pi-sigma linkages with LEU A: 701 and pi-alkyl linkages with VAL A: 816 and LEU A: 775.

**Figure 4:**
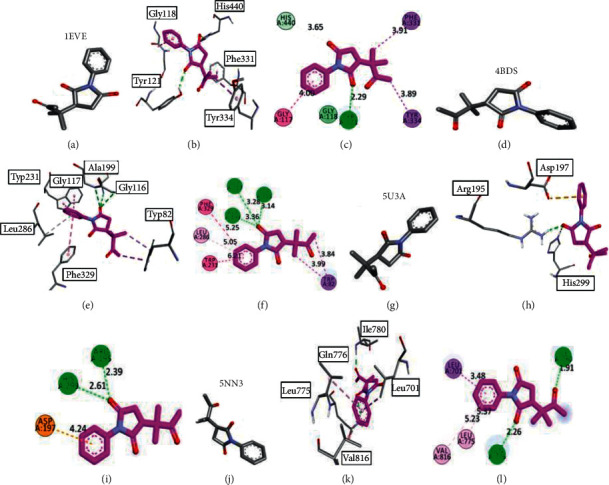
Docking 3-dimensional and 2-dimensional poses of ligand **MSJ10** inside the binding pockets of the targeted proteins. (a–c) indicate the best binding postures with acetylcholinesterase, (d–f) with butyrylcholinesterase, (g–i) with *α*-amylase, and (j–l) with *α*-glucosidase.

The protein-ligand complex of the synthesized chemical moiety **MSJ10** with all the targeted proteins including acetylcholinesterase, butyrylcholinesterase, *α*-amylase, and *α*-glucosidase displayed interesting features with binding affinities of −9.5 Kcal/mol, −9.1 Kcal/mol, −8.2 Kcal/mol, and −8.8 Kcal/mol, respectively Figure 4.

**Figure 5:**
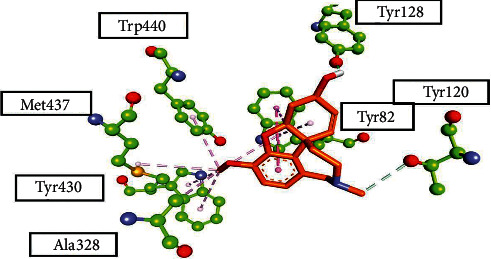
Three-dimensional visualization of the standard drug galantamine, inside the active binding site of butyrylcholinesterase PDB ID: 4BDS with amino acid (AA) residues' interaction shown.

This compound consists of 3,3-dimethylbutan-2-one side chain that provides a prominent interaction in the form of pi-sigma with PHE A: 331 and TYR A: 334 in the case of acetylcholinesterase. Conventional hydrogen bonds were formed with TYR A: 121 through the cyclopentane-1,3-dione structure. This complex established three conventional hydrogen bonds in butyrylcholinesterase with GLY A: 117, Gly A: 116, and ALA A: 119 through the cyclopentane-1,3-dione ring. The aromatic ring in this ligand formed pi-pi T-shaped linkages with PHR A: 329 and TRP A: 231, and pi-alkyl linkages with LEU A: 286 with a bond length of 5.25 Å, 6.21 Å, and 5.05 Å, respectively. Furthermore, TRP A: 82 formed pi-sigma linkages with 3,3-dimethylbutan-2-one. Further visualizing the binding affinity and interaction of this compound in *α*-amylase, it formed two conventional hydrogen bonds with ARG A: 195 and HIS A: 299 through the cyclopentane-1,3-dione ring. Pi-anion linkage provided more stability through ASP A: 197 interaction with an aromatic ring. This structural unit formed conventional hydrogen bonds with ILE A: 780 and GLN A: 776 through the side chain 3,3-dimethylbutan-2-one in the case of the *α*-glucosidase macromolecular structure. The interaction of the standard drug galantamine with the active site of butyrylcholinesterase has been elaborated in Figure 5. It gave the binding energy −9.6 Kcal/mol in its best pose. The interacting amino acid (AA) residues were TRP A: 82, THR A: 120, TYR: A 128, ALA A: 328, TRP A: 430, MET A: 437, and TRP A: 440. This interaction showed the important binding amino acid (AA) residues of the targeted macromolecule interacting with the standard drug that played an important role in initializing the response. This butyrylcholinesterase inhibition by the standard drug galantamine provides promising information that is used to compare the results with the synthesized succinimide derivatives.

## 4. Discussion

In pharmacological research, the most interesting fields are the discovery and development of multi-target drug-able moieties. Oxygen is an important component of the aerobic life, but it also has a negative effect on our health by initiating the development of free radicals like ROS (reactive oxygen species), which are responsible for diseases like cancer, diabetes, inflammation, neurodegenerative disorders (Alzheimer's and dementia), ulcers, immune suppression, aging, and atherosclerosis [38, 39]. The most common free radicals are lipid peroxyl, hydroxyl, nitric oxide, and superoxide. On the other hand, the most frequent non-free radicals are hydrogen peroxide and singlet oxygen [40]. However, our immune systems protect us from all these free radicals using the anti-oxidant defense system that slows the formation of free radicals while another system generates chain-breaking antioxidants to stabilize and scavenge free radicals [41, 42]. However, when the production of free radicals increases from the incompetency of the body's defense mechanisms, then severe tissue damage occurs [43]. Therefore, those drugs with potential free radical scavenging effects are useful in the treatment and prevention of various disorders [44]. Antioxidant substances are identified to possess biochemical effects on different pathways, including hydrogen abstraction for long-term, peroxide breakdown, chain initiation inhibition, radical scavenging, metal ion chelation, and reductive capacity [24, 45]. Therefore, numerous techniques have been suggested for the determination of the anti-oxidant activity. The most commonly used method to measure the scavenging capability of free radicals is the DPPH method [46]. Antioxidants scavenge DPPH radicals by giving hydrogen as a result of decrease in DPPH-H. After reduction, the color changes ultimately from purple to yellow, which is counted by evaluating the absorbance of the compounds at 571 nm wavelength [47]. In the ABTS test, the anti-oxidant capacity of the test sample was used to inhibit the oxidation of ABTS into ABTS^++^ radical cation [48].

The anti-cholinesterase enzyme presents a striking targeting moiety for normal drugs as well as for finding the mechanism's base inhibitors it plays a part in the breakdown of neurotransmitters like acetylcholine. The synthesis of AChE inhibitors presents an efficient approach to diagnose the mental symptoms of AD (Alzheimer disorder) and other promising therapeutic applications in the management of ataxia, senile dementia, and Parkinson's disease [49]. Furthermore, the compounds displayed substantial anti-cholinesterase scavenging activity, approximately halving the substrate breakage via human cholinesterase [50]. All the synthesized compounds include some stage of inhibitory potential against BChE and AChE. A low IC_50_ of the compounds displayed virtuous enzyme inhibition. **MSJ10, MSJ2,** and **MSJ7** had the final IC_50_ value, representing that they have good inhibition of the enzymes.

The in vitro *α*-amylase inhibition of the tested compounds was evaluated [51]. However, the enzyme *α*-amylase is present in pancreatic juice and saliva which can convert larger polysaccharides into smaller particles [52]. Similarly, the enzyme *α*-glucosidase was present in the small intestine which can convert disaccharides into monosaccharides. The metabolism of carbohydrates was delayed due to the inhibitory action on *α*-glucosidase and *α*-amylase, which decreased the postprandial blood glucose level but also has adverse drug reactions (ADRs) like diarrhea and intestinal problems [53, 54]. At present, acarbose was the drug of choice to delay the metabolism of carbohydrates by inhibiting the enzyme and also by decreasing postprandial blood glucose level, but it has ADRs like intestinal disorders and diarrhea [55, 56]. The *α*-amylase inhibition effect of **MSJ10** shows an IC_50_ value of 16.62 *µ*M and MSJ9 has 27.24 *µ*M. For *α*-glucosidase, **MSJ10** shows an IC_50_ of 28.04 *µ*M while **MSJ9** exhibited 32 *µ*M. The results are shown in Table 3.

## 5. Conclusions

In conclusion, we synthesized ketone succinimide derivatives. All these molecules were formed in a single step with excellent isolated produces. The finding of the current research work shows the devastating AChE, BChE, *α*-amylase, and *α*-glucosidase potentials of these compounds and reveals their central role in AD and diabetes. Furthermore, these compounds also scavenge ABTS and DPPH free radicals. Due to the background information and present studies of succinimide derivatives, it may be assumed that compound **MSJ10** is a potentially active compound as compared to the other derivatives and could be a significant drug against DM (diabetes mellitus) and AD (Alzheimer's disease), subsequently going through additional screening and evaluations.

## Figures and Tables

**Table 1:** DPPH and ABTS free radicals scavenging IC_50_ values of the synthesized compounds.

Compound	IC_50_ (*μ*M)	IC_50_ (*μ*M)
**MSJ1**	34.57	29.41
**MSJ2**	2.59	7.32
**MSJ3**	33.47	24.04
**MSJ4**	31.96	48.71
**MSJ5**	39.01	51.24
**MSJ6**	44.86	55.11
**MSJ7**	31.44	36.27
**MSJ8**	29.23	25.19
**MSJ9**	53.02	38.10
**MSJ10**	2.52	3.29
**Ascorbic acid**	6.25	4.66

**Table 2:** IC_50_ values of the synthesized compounds against the anti-cholinesterase inhibitory assay.

Compound	IC_50_ (*μ*M)	IC_50_ (*μ*M)
**MSJ1**	13.60	31.26
**MSJ2**	8.73	11.26
**MSJ3**	24.32	39.39
**MSJ4**	19.49	17.31
**MSJ5**	20.28	11.62
**MSJ6**	24.30	20.35
**MSJ7**	13.07	6.34
**MSJ8**	14.15	41.90
**MSJ9**	27.24	17.43
**MSJ10**	4.97	10.72
**Galantamine**	0.762	6.31

**Table 3:** In vitro anti-diabetic inhibitory activity of the synthesized compounds.

Compound	IC_50_ (*μ*M)	IC_50_ (*μ*M)
**MSJ1**	149.35	144.48
**MSJ2**	94.73	86.46
**MSJ3**	173.89	93.01
**MSJ4**	96.90	84.99
**MSJ5**	50.98	27.29
**MSJ6**	155.59	127.34
**MSJ7**	64.87	55.62
**MSJ8**	113.38	76.26
**MSJ9**	32.00	27.24
**MSJ10**	28.04	16.62
**Acarbose**	9.76	3.86

**Table 4:** Binding energy scoring of the synthesized ligands (**MSJ 1-10**) with targeted proteins.

Ligands	Binding energies (Kcal/mol)			
	Acetylcholinesterase	Butyrylcholinesterase	*α*-amylase	*α*-Glucosidase
	1EVE	4BDS	5U3A	5NN3
**MSJ1**	−6.5	−6.7	−7.1	−7.4
**MSJ2**	−8.7	−8.9	−8.2	−8.5
**MSJ3**	−6.9	−7.2	−7.4	−7.8
**MSJ4**	−7.5	−7.8	−7.1	−7.2
**MSJ5**	−6.9	−6.5	−6.5	−6.6
**MSJ6**	−7.1	−7.5	−7.4	−7.8
**MSJ7**	−7.4	−7.5	−7.4	−7.7
**MSJ8**	−6.9	−6.5	−6.4	−6.8
**MSJ9**	−6.6	−6.6	−6.7	−6.7
**MSJ10**	−9.5	−9.1	−8.2	−8.8
**Galantamine**	−9.9	−9.6	—	—
**Acarbose**	—	—	−9.1	−9.5

## Data Availability

The data used to support the findings of the study can be obtained from the corresponding author upon request.

## Supplementary Materials

Supplementary Materials The spectral data of the synthesized compounds (Figure S1–S10) as well as various pharmacological activities like anti-oxidant (Figure S11, S12), anti-cholinesterase (Figure S13, S14), and anti-diabetic percent inhibition details (Figure S15, S16) are provided in the Supporting Information.
