# Supplementary material for: Succinimide Derivatives as Antioxidant Anticholinesterases, Anti-α-Amylase, and Anti-α-Glucosidase: In Vitro and In Silico Approaches
Source: Evid Based Complement Alternat Med. 2022 Jul 30;2022:6726438. doi: 10.1155/2022/6726438 (PMC9356783; doi:10.1155/2022/6726438)
Supplement: Supplementary Materials — The spectral data of the synthesized compounds (Figure S1–S10) as well as various pharmacological activities like anti-oxidant (Figure S11, S12), anti-cholinesterase (Figure S13, S14), and anti-diabetic percent inhibition details (Figure S15, S16) are provided in the Supporting Information. [file 6726438.f1.doc]

# Succinimide Derivatives as Antioxidant Anticholinesterases, anti-α-amylase, and anti-α-glucosidase: In-vitro and In-silico Approaches

Osama M. Alshehri^1^([usamah2012@gmail.com](mailto:usamah2012@gmail.com))

Mater H. Mahnashi^2^ ([matermaha@gmail.com](mailto:matermaha@gmail.com))

Abdul Sadiq*^3^ ([sadiquom@yahoo.com](mailto:sadiquom@yahoo.com))

Rehman Zafar^4^ ([rehmanzafar016@gmail.com](mailto:rehmanzafar016@gmail.com))

Muhammad Saeed Jan*^5^ ([saeedjanpharmacist@gmail.com](mailto:saeedjanpharmacist@gmail.com))

Farhat Ullah^3^ ([farhataziz80@hotmail.com](mailto:farhataziz80@hotmail.com))

Mohammed Ali Alshehri^6^ ([maalshehri@nu.edu.sa](mailto:maalshehri@nu.edu.sa))

Saleh Alshamrani^1^([saalshamrani@nu.edu.sa](mailto:SAALSHAMRANI@NU.EDU.SA))

Elhashimi E Hassan^1^ ) [alhashimihassan2018@gmail.com](mailto:alhashimihassan2018@gmail.com))

^1^Department of Clinical Laboratory Sciences, College of Applied Medical Sciences, Najran University, Najran, Saudi Arabia.^2^Department of Pharmaceutical Chemistry, College of Pharmacy, Najran University, Najran, Saudi Arabia.^3^Department of Pharmacy, Faculty of Biological Sciences, University of Malakand, Chakdara, KP 18000 Dir (L), Pakistan. ^4^Riphah Institute of Pharmaceutical Sciences, Riphah International University, Islamabad,44000, Pakistan. ^5^Department of Pharmacy, University of Swabi, KP, Pakistan. ^6^Department of Medical Genetics, Collage of Applied Medical Sciences, Najran University, Najran, Saudi Arabia

**Corresponding authors:** *Dr. Muhammad Saeed Jan, Assistant Professor, Department of Pharmacy, University of Swabi, KP, Pakistan, Contact: +92(0)315 3109 610, Email: [saeedjanpharmacist@gmail.com](mailto:saeedjanpharmacist@gmail.com), *Dr. Abdul Sadiq, Tenured Associate Professor, Department of Pharmacy, Faculty of Biological Sciences, University of Malakand, Chakdara, 18000 Dir (L), KP, Pakistan, Contact: +92(0)301 2297 102, Email: [sadiquom@yahoo.com](mailto:sadiquom@yahoo.com),

**Scheme S1:** Synthesis of the compounds **MSJ-1** to **MSJ-10**.

## 3-(2-oxocyclohexyl)-1-phenylpyrrolidine-2,5-dione (MSJ-1)

The total duration of reaction completion was 23 h and the color of the obtained product was white solid with 90 % isolated yield. The R_f_ value in *n*-hexane and ethyl acetate (3:1) was calculated as 0.41.^1^H NMR (400 MHz, CDCl_3_) (ppm): 1.51-1.82 (m, 3H), 1.96-2.04 (m, 1H), 2.07-2.22 (m, 2H), 2.31-2.48 (m, 2H), 2.52-2.67 (m, 1H), 2.82-2.90 (m, 1H), 3.02-3.12 (m, 1H), 3.19-3.33 (m, 1H), 7.24-7.33 (m, 2H), 7.35-7.40 (m, 1H), 7.44-7.50 (m, 2H).^13^C NMR (100 MHz, CDCl_3_) (ppm): 22.08, 25.41, 29.26, 30.13, 31.39, 32.62, 38.46, 40.42, 41.71, 50.11, 52.30, 126.50, 126.52, 128.68, 129.20, 131.94, 175.09, 177.44, 210.19. HPLC purity = 95.2 %, TR = 10.0 min. LC-MS found for C16H17NO3 (m/z) = 272.2 [M+H]. Analysis calcd (%): C, 70.83; H, 6.32; N, 5.16. Found (%): C, 71.03; H, 6.29; N, 5.21.


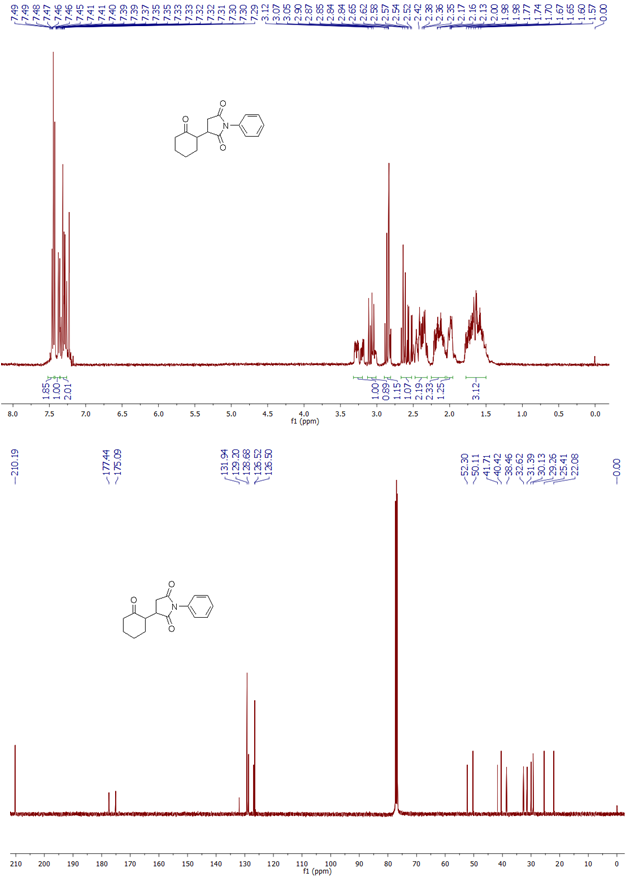


Figure S1: ^1^HNMR & ^13^CNMR spectrum of MSJ-1

## 1-benzyl-3-(2-oxocyclohexyl) pyrrolidine-2,5-dione (MSJ-2)

The duration for the reaction completion was 24 h and the color of the obtained product was half white with 69 % isolated yield. The R_f_ value in *n*-hexane and ethyl acetate (3:1) was calculated as 0.45.

^1^H NMR (400 MHz, CDCl_3_) (ppm): 1.49-1.79 (m, 3H), 1.83-2.01 (m, 2H), 2.12-2.26 (m, 1H), 2.33-2.63 (m, 3H), 2.80-2.99 (m, 2H), 3.02-3.16 (m, 1H), 4.65 (d, J = 7.38 Hz, 2H), 7.26-7.38 (m, 5H).^13^C NMR (100 MHz, CDCl_3_) (ppm): 24.03, 26.22, 27.99, 32.03, 32.97, 39.54, 40.18, 40.88, 41.80, 49.99, 51.52, 126.99, 127.61, 128.32, 129.09, 129.81, 133.87, 175.16, 178.28, 210.16.HPLC purity = 96.4 %, TR = 10.9 min. LC-MS found for C17H19NO3 (m/z) = 286.2 [M+H]. Analysis calcd (%): C, 71.56; H, 6.71; N, 4.91; Found (%): C, 71.43; H, 6.73; N, 4.94.


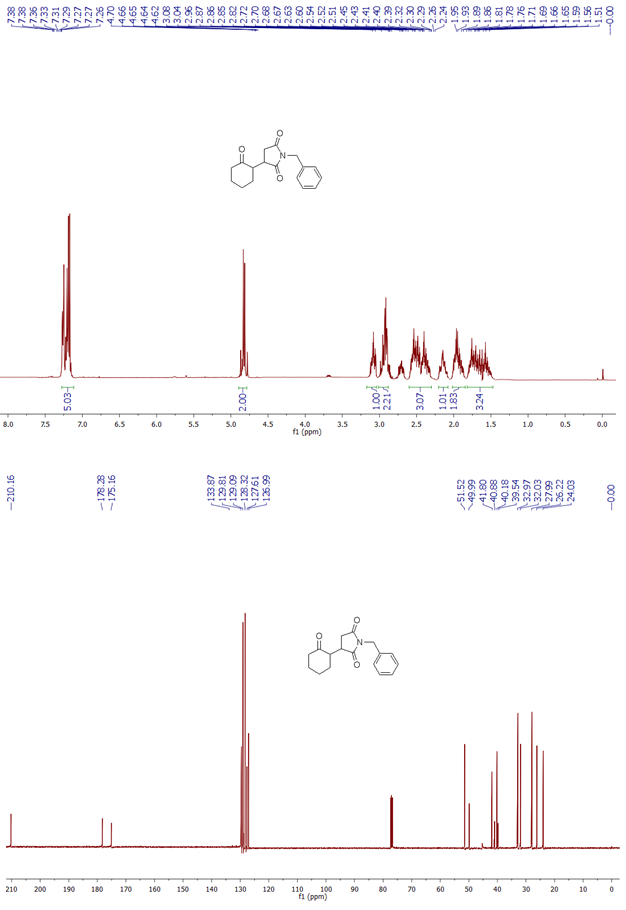


Figure S2: ^1^HNMR & ^13^CNMR spectrum of MSJ-2

## 1-(4-bromophenyl)-3-(2-oxocyclohexyl) pyrrolidine-2,5-dione (MSJ-3)

The total duration for the reaction completion was 30 h and the color of the obtained product was yellowish with 75 % isolated yield. The R_f_ value in *n*-hexane and ethyl acetate (4:1) was calculated as 0.43.

^1^H NMR (400 MHz, CDCl_3_) (ppm): 1.64-1.77 (m, 3H), 2.06-2.21 (m, 4H), 2.47-2.52 (m, 3H), 1.96-3.02 (m, 2H), 7.24-7.28 (m, 2H), 7.59-7.64 (m, 2H).^13^C NMR (100 MHz, CDCl_3_) (ppm): 23.15, 24.29, 27.37, 28.13, 30.69, 31.46, 32.49, 33.76, 41.82, 42.48, 43.56, 54.08, 54.77, 124.12, 129.20, 131.03, 133.66, 175.56, 177.81, 211.58. HPLC purity = 98.1 %, TR = 13.9 min. LC-MS found for C16H16BrNO3 (m/z) = 350.1 [M+H]. Analysis calcd (%): C, 54.87; H, 4.61; N, 4.00 %. Found (%): C, 54.97; H, 4.60; N, 4.03.


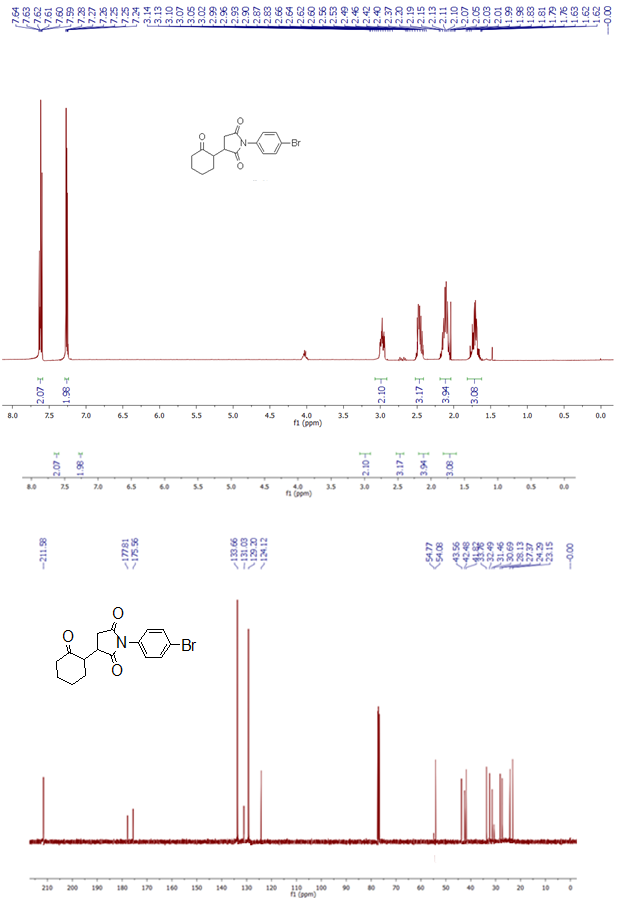


**Figure S3:** ^1^HNMR & ^13^CNMR spectrum of **MSJ-3**

## 3-(5-methyl-2-oxocyclohexyl)-1-phenylpyrrolidine-2,5-dione (MSJ-4)

The reaction was fully completed in 24 h. The color of the obtained compound was white solid with 79 % isolated yield. The R_f_ value was 0.49 while *n*-hexane and ethyl acetate was used as eluent with ratio of 4:1.

^1^H NMR (400 MHz, CDCl_3_) (ppm): 0.95-0.96 (m, 1H), 1.14-1.20 (m, 3H), 1.26-1.44 (m, 1H), 1.61-1.83 (m, 1H), 1.87-2.03 (m, 2H), 2.16-2.36 (m, 2H), 2.43-2.81 (m, 2H) 2.94-2.97 (m, 1H), 3.03-3.15 (m, 1H), 7.19-7.27 (m, 2H), 7.31-7.33 (m, 1), 7.38-7.41 (m, 2H). ^13^C NMR (100 MHz, CDCl_3_) (ppm): 17.67, 17.70, 21.35, 21.37, 26.88, 26.93, 32.00, 32.40, 33.37, 33.60, 34.92, 35.24, 35.72, 37.13, 37.18, 37.41, 38.18, 41.02, 41.22, 41.34, 46.15, 47.47, 51.30, 126.76, 126.78, 126.93, 128.74, 129.29, 132.25, 132.54, 175.83, 175.88, 178.64, 178.74, 210.45, 210.85. HPLC purity = 96.5 %, TR = 11.2 min. LC-MS found for C17H19NO3 (m/z) = 286.5 [M+H]. Analysis calcd (%): C, 71.56; H, 6.69; N, 4.91. Found (%): C, 71.76; H, 6.71; N, 4.93.


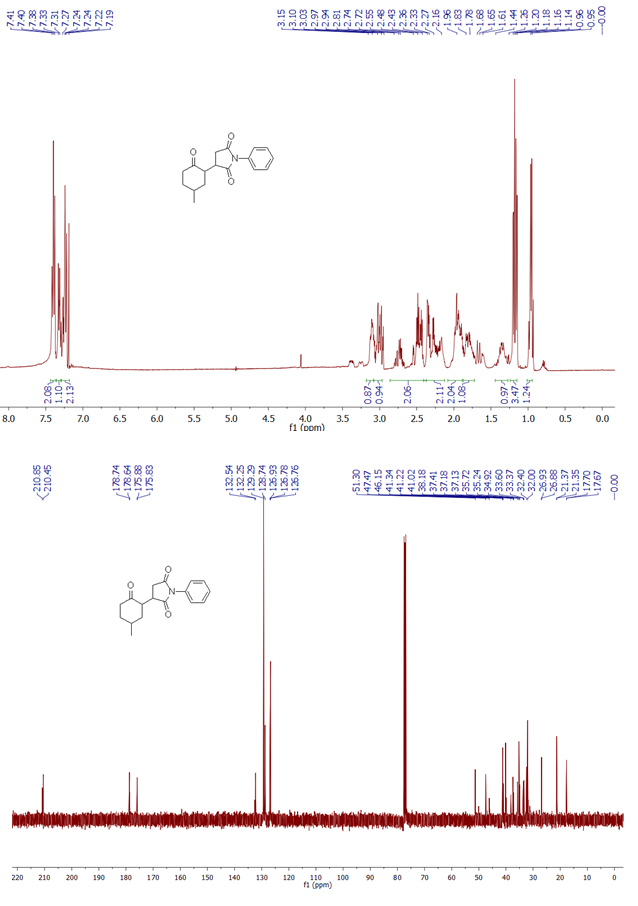


Figure S4: ^1^HNMR & ^13^CNMR spectrum of MSJ-4

## 1-benzyl-3-(5-methyl-2-oxocyclohexyl)pyrrolidine-2,5-dione (MSJ-5)

The total duration for the reaction completion was 24 h and color of the obtained compound was yellowish with 63 % isolated yield. The R_f_ value in *n*-hexane and ethyl acetate (4:1) was calculated as 0.46.

^1^H NMR (400 MHz, CDCl_3_) (ppm): 1.00 (d, J = 6.58 Hz, 3H), 1.16-1.26 (m, 1H), 1.32-1.45 (m, 2H), 1.83-2.02 (m, 4H), 2.24-2.36 (m, 3H), 2.63-3.08 (m, 1H), 4.62-4.73 (m, 2H), 7.23-7.39 (m, 5H). ^13^C NMR (100 MHz, CDCl_3_) (ppm): 17.77, 17.83, 21.21, 21.30, 26.82, 26.95, 31.79, 32.32, 32.71, 32.92, 34.97, 35.22, 37.24, 37.42, 39.94, 41.04, 41.17, 41.38, 42.56, 45.52, 46.76, 50.56, 99.54, 100.07, 127.84, 128.05, 128.63, 128.69, 128.77, 128.83, 136.07, 176.42, 176.54, 179.43, 210.18, 212.54. HPLC purity = 95.7 %, TR = 12.1 min. LC-MS found for C18H21NO3 (m/z) = 300.2 [M+H]. Analysis calcd (%): C, 72.22; H, 7.07; N, 4.68. Found (%): C, 72.01; H, 7.09; N, 4.71.


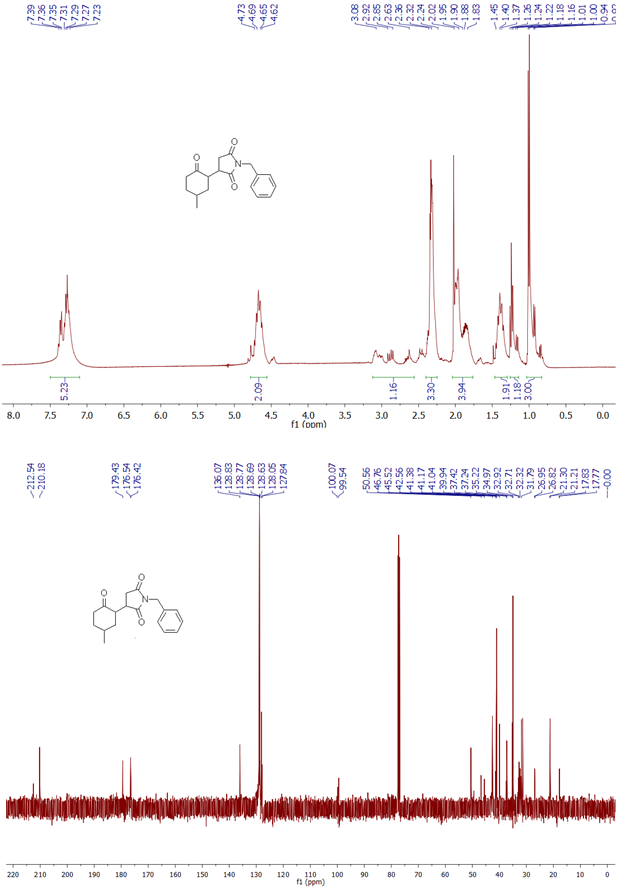
Figure S5: ^1^HNMR & ^13^CNMR spectrum of MSJ-5

## 3-(4-oxotetrahydro-2H-pyran-3-yl)-1-phenylpyrrolidine-2,5-dione (MSJ-6)

The total reaction completion time was 19 h and color of the obtained product was half white having 73 % isolated yield. The R_f_ value in chloroform and methanol(6:1) was calculated as 0.44.

^1^H NMR (400 MHz, CDCl_3_) (ppm): 2.33-2.41 (m, 1H), 2.71-3.19 (m, 5H), 3.52-3.78 (m, 2H), 4.26-4.75 (m, 2H), 7.25-7.48 (m, 5H).^13^C NMR (100 MHz, CDCl_3_) (ppm): 31.47, 32.30, 36.97, 41.68, 43.14, 51.23, 53.11, 66.62, 69.01, 70.22, 126.31, 128.73, 129.21, 129.89, 174.96, 177.64, 204.01.HPLC purity = 98.4 %, TR = 6.3 min. LCMS found for C15H15NO4 (m/z) = 274.1 [M+H]. Analysis calcd (%): C, 65.92; H, 5.53; N, 5.13. Found (%): C, 65.73; H, 5.52; N, 5.15.


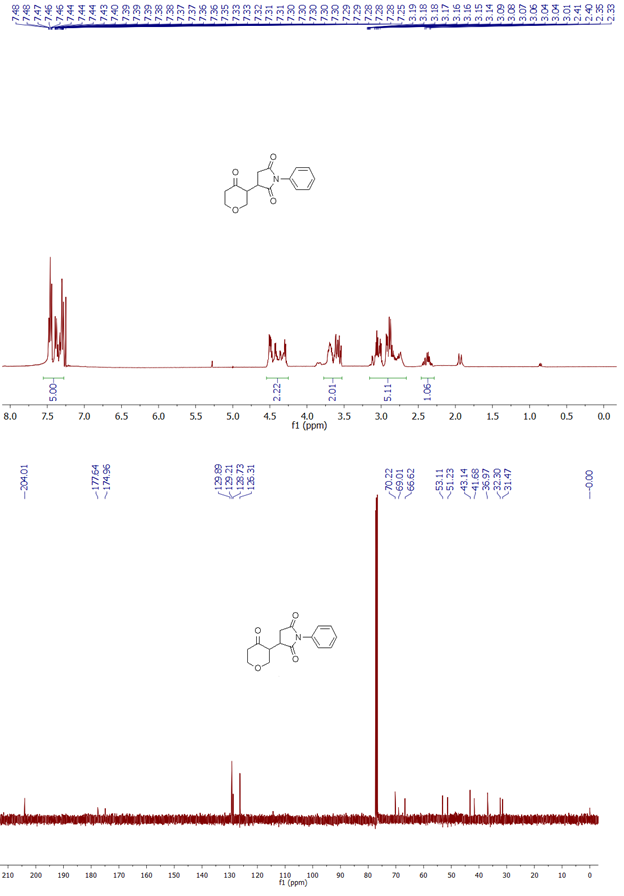


Figure S6: ^1^HNMR & ^13^CNMR spectrum of MSJ-6

## 3-(2-oxocycloheptyl)-1-phenylpyrrolidine-2,5-dione (MSJ-7)

The total duration of reaction completion was 18 h and the color of the obtained product was white with 78 % isolated yield. The R_f_ value in *n*-hexane and ethyl acetate (4:1) was calculated as 0.53.

^1^H NMR (400 MHz, CDCl_3_) (ppm): 1.23-1.99 (m, 6H), 2.00-2.27 (m, 2H), 2.61-2.75 (m, 2H), 2.75-3.01 (m, 2H), 3.22-3.38 (m, 1), 3.42-3.55 (m, 1H), 7.24-7.27 (m, 1H), 7.32-7.35 (m, 1H), 7.37-7.41 (m, 1H), 7.45-7.50 (m, 2H).^13^C NMR (100 MHz, CDCl_3_) (ppm): 25.22, 28.94, 30.31, 30.51, 30.66, 31.06, 31.70, 33.39, 33.55, 34.11, 38.64, 53.19, 54.14, 127.06, 127.30, 128.21, 129.77, 130.15, 133.66, 134.61, 174.91, 175.12, 177.27, 213.86, 214.03.HPLC purity = 97.1 %, TR = 13.5 min. LC-MS found for C17H19NO3 (m/z) = 286.1 [M+H]. Analysis calcd (%): C, 71.56; H, 6.71; N, 4.91. Found (%): C, 71.70; H, 6.72; N, 4.88.


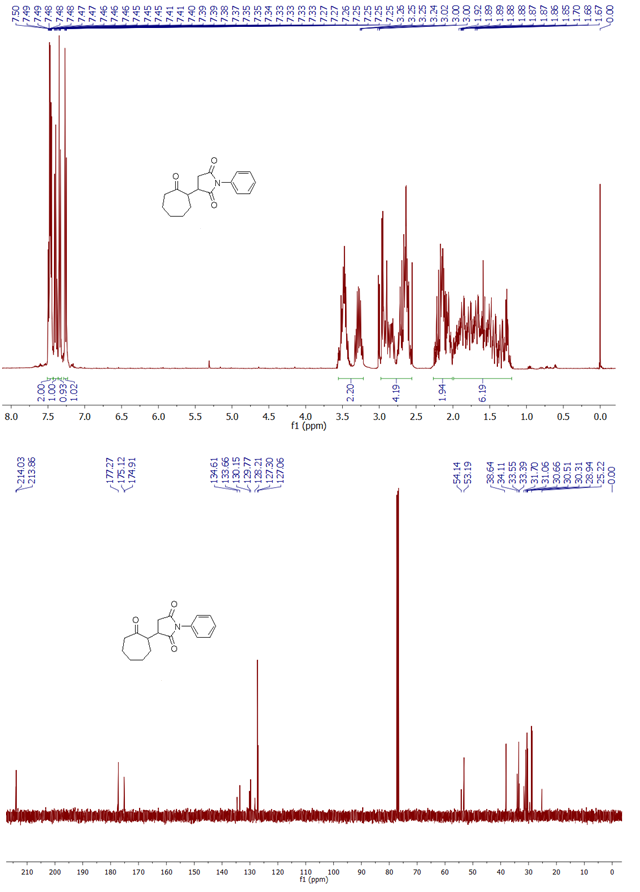


Figure S7: ^1^HNMR &  ^13^CNMR spectrum of MSJ-7

## 3-(2-oxocyclopentyl)-1-phenylpyrrolidine-2,5-dione (MSJ-8)

The total duration of reaction completion was20 h and the color of the obtained product was yellowish with 74 % isolated yield. The R_f_ value in *n*-hexane and ethyl acetate (2:1) was calculated as 0.40.

^1^H NMR (400 MHz, CDCl_3_) (ppm): 1.82-1.95 (m, 2H), 2.06-2.17 (m, 2H), 2.18-2.26 (m, 2H), 2.36-2.45 (m, 1H), 2.95 (dd, *J* = 5.26 and 18.39 Hz, 1H), 2.82-2.89 (m, 1H), 2.99 (dd, *J* = 9.66 and 18.39 Hz, 1H), 3.45 (ddd, *J* = 8.43, 5.26 and 3.17 Hz, 1H), 7.23-7.27 (m, 2H), 7.36-7.41 (m, 1H), 7.44-7.49 (m, 2H).^13^C NMR (100 MHz, CDCl_3_) (ppm): 23.69, 25.59, 30.68, 37.96, 40.01, 51.08, 127.07, 128.92, 129.81, 132.78, 176.88, 179.82, 216.19.HPLC purity = 97.3 %, TR = 9.1 min. LC-MS found for C15H15NO3 (m/z) = 258.1 [M+H]. Analysis calcd (%): C, 70.02; H, 5.88; N, 5.44. Found (%): C, 70.21; H, 5.86; N, 5.47.


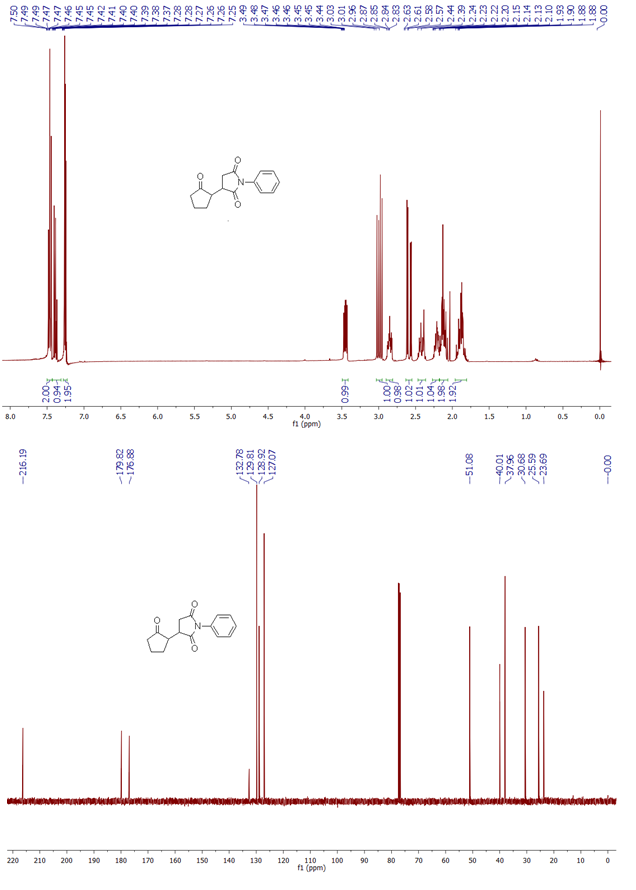
Figure S8: ^1^HNMR & ^13^CNMR spectrum of MSJ-8

## 3-(2-oxopropyl)-1-phenylpyrrolidine-2,5-dione (MSJ-9)

The total duration of reaction completion was 24 h and the color of the obtained product was yellowish with 76 % isolated yield. The R_f_ value in *n*-hexane and ethyl acetate (2:1) was calculated as 0.43.

^1^H NMR (400 MHz, CDCl_3_) (ppm): 2.18 (s, 3H), 2.52-2.58 (m, 1H), 2.99-2.21 (m, 4H), 2.26-7.49 (m, 5H).^13^C NMR (100 MHz, CDCl_3_) (ppm): 29.94, 35.15, 36.47, 41.96, 126.31, 128.25, 129.09, 131.27, 175.18, 178.31, 205.59.HPLC purity = 98.1 %, TR = 6.2 min.


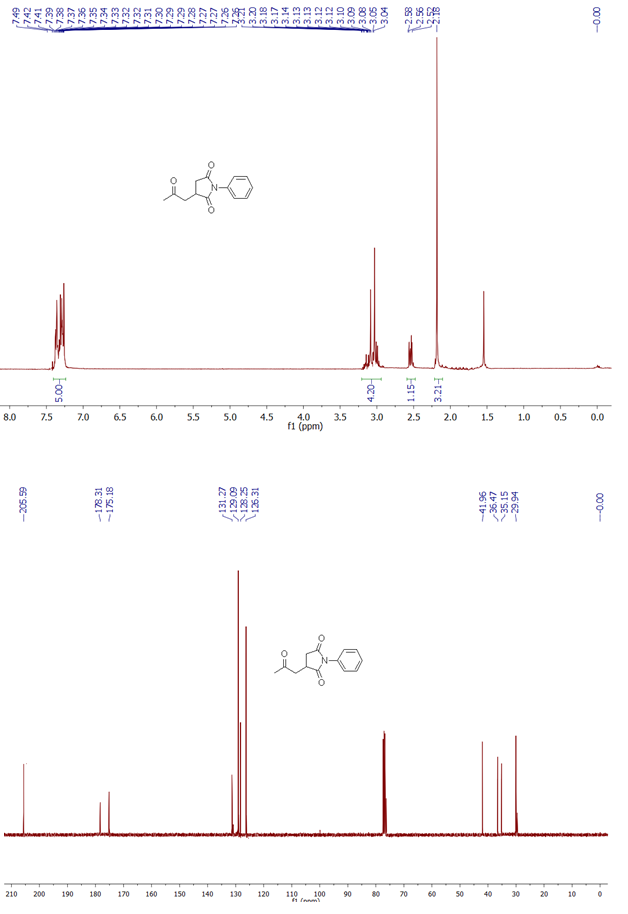


Figure S9: ^1^HNMR & ^13^CNMR spectrum of MSJ-9

## 3-(2-methyl-3-oxobutan-2-yl)-1-phenylpyrrolidine-2,5-dione (MSJ-10)

The total duration of reaction completion was 24 h and the color of the obtained product was yellow solid with 70% isolated yield. The R_f_ value in *n*-hexane and ethyl acetate (4:1) was calculated as 0.51.

^1^H NMR (400 MHz, CDCl_3_) (ppm): 1.12 (s, 3H), 1.35 (s, 3H), 2.15 (s, 3H), 2.52 (dd, *J* = 5.40 and 9.39Hz, 1H), 2.94 (dd, J = 9.39 and 18.31 Hz, 1H), 3.2 (dd, *J* = 5.40 and 18.31 Hz, 1H), 7.25-7.28 (m, 2H), 7.29-7.33 (m, 1H), 7.34-7.39 (m, 2H).^13^C NMR (100 MHz, CDCl_3_) (ppm): 22.23, 24.09, 25.68, 44.38, 50.63, 128.01, 128.67, 128.75, 133.05, 175.20, 176.95, 212.58.HPLC purity = 98.5 %, TR = 6.2 min.

**
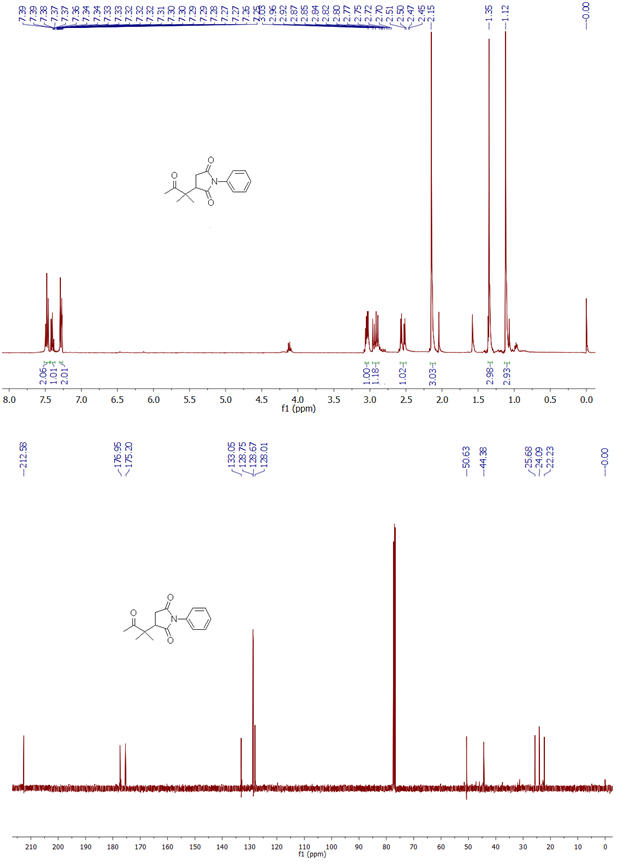
**

Figure S10: ^1^HNMR & ^13^CNMR spectrum of MSJ-10

**Results of Anti-oxidant (ABTS) assay:**

**Figure S11:** Antioxidant potential of the synthesized compounds using ABTS assay. Values represent % radical scavenging (mean ± SEM) of three replicates, Data were analyzed by Two-way ANOVA followed by Bonferoni post test; Values significantly different as compare to positive control, *P < 0.05, **P < 0.01***P < 0.001, ns; not significant.

**Results of Anti-oxidant (DPPH) assay:**

**Figure S12:** Antioxidant potential of the synthesized compounds using DPPH assay. Values represent % DPPH free radical scavenging (mean ± SEM) of three replicates, Data were analyzed by Two-way ANOVA followed by Bonferoni post test; Values significantly different as compare to positive control, *P < 0.05, **P < 0.01***P < 0.001, ns; not significant.

**Results of anti-cholinesterase (AChE) assay:**

**Figure S13:** Anticholinesterase activity of the synthesized compounds using AChE assay. Values represent % AChE inhibition (mean ± SEM) of three replicates, Data were analyzed by Two-way ANOVA followed by Bonferoni post test; Values significantly different as compare to positive control, *P < 0.05, **P < 0.01***P < 0.001, ns; not significant.

**Results of anti-cholinesterase (BChE) assay:**

**Figure S14:** Anticholinesterase activity of the synthesized compounds using BChE assay. Values represent % BChE inhibition (mean ± SEM) of three replicates, Data were analyzed by Two-way ANOVA followed by Bonferoni post test; Values significantly different as compare to positive control, *P < 0.05, **P < 0.01***P < 0.001, ns; not significant.

**Results of anti-diabetic (α- Glucosidase) assay:**

**Figure S15:** Anti-diabetic activity of the synthesized compounds using α- glucosidase inhibitory assay. Values represent % enzyme inhibition (mean ± SEM) of three replicates, Data were analyzed by Two-way ANOVA followed by Bonferoni post test; Values significantly different as compare to positive control, *P < 0.05, **P < 0.01***P < 0.001, ns; not significant.

**Results of anti-diabetic (α- Amylase) assay:**

**Figure S16:** Anti-diabetic activity of the synthesized compounds using α- Amylase inhibitory assay. Values represent % enzyme inhibition (mean ± SEM) of three replicates, Data were analyzed by Two-way ANOVA followed by Bonferoni post test; Values significantly different as compare to positive control, *P < 0.05, **P < 0.01***P < 0.001, ns; not significant.
